# Postbiotic studies of mixed cultures of *Schleiferilactobacillus harbinensis LH991* and *Pichia kudriavzevii* B-5P produced b*y in vitro* rumen producing short-chain fatty acid

**DOI:** 10.14202/vetworld.2024.2694-2700

**Published:** 2024-11-30

**Authors:** Yetti Marlida, Tan Joo Shun, Syofyan Syofyan, Laily Rinda Ardani, Lili Anggraini

**Affiliations:** 1 Department of Animal Nutrition, Faculty of Animal Science, Andalas University, Limau Manis Campus, Padang, West Sumatra 25163, Indonesia; 2 Department of Bioprocess Technology, School of Industrial Technology, Universiti Sains Malaysia, Gelugor, Pulau Pinang, Malaysia; 3 Department of Pharmaceutics, Faculty of Pharmacy, Andalas University, Limau Manis Campus, Padang, West Sumatra 25163, Indonesia; 4 Doctoral Program, Faculty of Animal Science, Andalas University, Padang, West Sumatra 25163, Indonesia; 5 Research Organization of Agriculture and Food, National Research and Innovation Agency, Bogor, 16911, Indonesia

**Keywords:** digestibility, *in-vitro*, postbiotics, probiotics, short-chain fatty acids

## Abstract

**Background and Aim:**

Postbiotics are functional bioactive compounds or bioactive molecules with beneficial effects on health and functional activities in humans or livestock, produced by probiotic bacteria or yeast. Several postbiotics, including enzymes, short-chain fatty acids, amino acids, extracellular polysaccharides, microbial cell fragments, and teichoic acids, are currently being widely studied. This study aimed to explore the potential of secondary metabolites of *Schleiferilactobacillus harbinensis* *LH 991* and *Pichia kudriavzevii* *B-5P* as lactic acid bacteria (LAB) and yeast isolated from Budu (fermented fish) which can act as postbiotics through *in vitro* rumen fermentation.

**Materials and Methods:**

The method used a completely randomized design 5 × 4, with five treatments and four replications. The substrate diet consisted of 60% forage and 40% concentrate. The culture mixture was 1.3 × 10^11^ CFU/mL with a 50%:50% ratio of *S. harbinensis* *LH 991* and *P. kudriavzevii B-5P*. The inoculum concentrations used in this study were 0% (control), 1%, 2%, 3%, and 4%. Treatments are arranged based on differences in inoculum concentration as follows: T0: control (0%); T1: 1%; T2: 2%; T3: 3%; and T4: 4%.

**Results:**

The T4 group showed a significant increase (p < 0.01) in short-chain fatty acids (SCFA), including acetate, propionate, butyrate, valerate, isobutyrate, and isovalerate acids, compared with the other treatments. Meanwhile, T4 shows that there is no significant (p > 0.01) effect on *in vitro* digestibility (*in vitro* dry matter digestibility, *in vitro* organic matter digestibility, and *in vitro* crude fiber digestibility). However, a highly significant (p < 0.01) effect was on volatile fatty acid total, NH_3_, and microbial crude protein synthesis.

**Conclusion:**

It is concluded that the treatment with a 4% inoculum concentration (T4) containing a mixture of *S. harbinensis LH 991* and *P. kudriavzevii B-5P* as LAB and yeast isolated from Budu (fermented fish) in 50%:50% ratio increased SCFA and rumen fermentation significantly, whereas it did not affect *in vitro* digestibility.

## Introduction

The use of antibiotics is currently prohibited in various countries because it harms livestock and humans who consume livestock products. However, the search for safer and more beneficial antibiotic substitutes for livestock productivity is currently intensified [1]. Several substitutes for antibiotics that can be used for livestock productivity include probiotics, prebiotics, synbiotics, and postbiotics, which were recently discovered [2]. Postbiotics, which are the metabolic by-products of probiotics, have recently been preferred and have shown positive results [3]. Postbiotics have a positive impact like the use of probiotics, but without containing live microbial cells [4]. Postbiotics can improve intestinal health and inhibit pathogenic bacteria to optimize productivity and nutrient utilization [5]. Various types of postbiotic molecules include the secondary metabolites of live probiotic bacteria, such as organic acids, short-chain fatty acids (SCFA), cell-free supernatants, secreted proteins/peptides, amino acids, and bacteriocins [6, 7]. The previous studies have reported that postbiotics can be used as feed additives in monogastric livestock such as poultry and pigs to improve productivity and health [3, 4]. Postbiotics are considered easier to apply and handle. Postbiotics have similar effects to probiotics without the live cells contained [6]. Marlida *et al*. [8] found that probiotic yeast has potential as a probiotic isolated from fermented fish (Budu), while Susalam *et al*. [9] discovered various lactic acid bacteria (LAB) isolates from Budu have potential as a consortium probiotic for broilers, one of them being *Schleiferilactobacillus harbinensis LH 991*. A previous study by Marlida *et al*. [10] reported that local food sources, such as Budu, which is native to West Sumatra, contain a biodiversity of bacteria with various potential benefits. This research is a continuation study of Ardani *et al*. [11], who found that the candidate probiotics from Budu (fermented fish), which are *S. harbinensis LH 991* and *Pichia kudriavzevii B-5P*, can enhance nutrient digestibility and rumen characteristics in ruminants.

Postbiotics can contain various metabolites resulting in intermediate or final products in the metabolism of *Lactobacillus sp*., especially acetic and lactic acids, as well as antimicrobial peptides commonly known as bacteriocins [12, 13]. Postbiotics have a probiotic effect that lowers the pH in the intestine, increasing the lactic acid concentration of the bacterial population and reducing *Enterobacteriaceae* populations [13]. Postbiotics also improve growth performance and immune status, enhance the length of intestinal villi, and reduce the number of pathogens in broiler chickens [14], ruminants [15], laying hens [16], and pigs [17]. In several recent studies reported by Zhong *et al*. [18], the use of postbiotics in monogastric livestock has been widely used; however, information regarding the use of postbiotics in ruminant feed is still limited. Interaction of LAB with the rumen microorganisms improves fermentation and prevents the pathogen. LAB produces stable lactic acid in the rumen, allowing the microflora to adapt to the accumulation of lactic acid, increasing lactic-utilizing bacteria, and stabilizing rumen pH [15, 19]. In addition, yeast is a microbial culture that is commonly used in animal husbandry because it increases the production of volatile fatty acids (VFA) and rumen. A yeast can also stimulate the immune system of livestock [20]. Ji *et al*. [20] reported that *P. kudriavzevii* isolated from cow rumen showed extraordinary potential in biomass and cellulase production. Similar results can be achieved using postbiotics as feed additives for livestock. Therefore, it is necessary to evaluate the secondary metabolites produced by *S. harbinensis LH 991* and *P. kudriavzevii B-5P* through *in vitro* rumen fermentation.

The rumen is a natural bioreactor that allows probiotics to produce postbiotic metabolites [21]. Ardani *et al*. [11] have reported that the probiotics *S. harbinensis LH 991* and *P. kudriavzevii B-5P* produce the highest levels of VFA and NH_3_ in *in vitro* rumen; however, the results of secondary metabolites such as SCFA and various other metabolites have not been studied further. The administration of two probiotics such as *S. harbinensis LH 991* and *P. kudriavzevii B-5P* is expected to increase the levels of secondary metabolites, including SCFAs. Nataraj *et al*. [22] reported that added postbiotics are a complex mixture of metabolic products secreted by probiotics. Different probiotics provide different postbiotics.

This study aimed to explore the potential of secondary metabolites such as SCFAs through *in vitro* rumen fermentation from *S. harbinensis LH 991* and *P. kudriavzevii B-5P*, which can act as postbiotics.

## Materials and Methods

### Ethical approval

This study did not use live animals, so ethical approval was not required. Rumen fluid was obtained from a slaughterhouse of goats.

### Study period and location

This study was conducted from July to November 2023 at the Feed Industry Technology, Non-Ruminant Nutrition, and Ruminant Nutrition Laboratory, Faculty of Animal Science, Andalas University, Indonesia. SCFA content testing was performed at the Livestock Research Institute (Balitnak), Bogor, Indonesia.

### Inoculum preparation

The two strains of *S. harbinensis LH 991* and *P. kudriavzevii B-5P* used in this research were obtained from the laboratory collections of the Food Industry Technology Laboratory, Department of Animal Nutrition, Andalas University. The stock inoculum of LAB was inoculated in MRS Broth medium (Merck KGaA, Germany) in 10 mL. Then, cells were incubated under anaerobic conditions for 24–48 h at 37°C. The yeast inoculum was prepared in Yeast Peptone Dextrose medium (Merck KGaA, Germany) in 10 mL and incubated at 35°C–37°C for 24–48 h. Inoculum of *S. harbinensis LH 991* and *P. kudriavzevii B-5P* mixtures with a composition of 50%:50% ratio.

### Experimental design

The method used a completely randomized design 5 × 4, with five treatments and four replications. The substrate diet consisted of 60% forage and 40% concentrate. The culture mixture was 1.3 × 10^11^ CFU/mL with 50%:50% ratio of *S. harbinensis* *LH 991* and *P. kudriavzevii B-5P*. Treatments were arranged based on differences in inoculum concentration as follows: T0: control (0%); T1: 1%; T2: 2%; T3: 3%; and T4: 4%. After these processes, the samples were processed for nutrient ingredient analysis and *in vitro* evaluation.

### Nutrient ingredient analysis

The contents of feed ingredients of all treatments, including dry matter, organic matter, crude fat, crude fiber, crude protein, and ash, were analyzed using proximate analysis [23]. The results of the analysis are presented in Table-1.

**Table-1:** Nutrient ingredients of experimental diets (% DM).

Nutrient ingredients	Content (%)
Forage: Concentrate	60:40
Chemical compounds	
DM	83.67
Organic matter	87.58
Crude fiber	26.55
Crude fat	1.56
Crude protein	27.20
Total digestible nutrient	63.66
Ash	14.85

Analysis results from the Animal Biotechnology Laboratory of the Faculty of Animal Science at Andalas University (2023). DM=Dry matter

### *In vitro* fermentation and parameter measurement

This study followed Tilley and Terry’s method [24] to conduct rumen *in vitro* incubation. In total, 2.5 g of substrate was incubated with 200 mL of buffer solution and 50 mL of rumen fluid in a fermenter tube. Rumen fluid was obtained from a slaughterhouse of goats with an average body weight ± 45 kg. Once incubation was complete, each tube was placed in a tub filled with ice water to stop microbial action, after which the rumen pH was measured.

The contents of the fermenter tube were separated into the supernatant and residue using a centrifuge at 4°C and a speed of 1509× *g* for 5 min. The liquid part or supernatant was stored in a −18°C freezer until further analysis of rumen characteristics, including pH, NH_3_, total VFA, microbial protein synthesis, and SCFA. Meanwhile, the solid residue was filtered using filter paper and dried for 24 h in an oven at 60°C. The nutrient content of dried residue was determined following proximate [23]. The digestibility of feed nutrients was calculated following Marlida *et al*. [25]. SCFA content was measured using a gas chromatograph (GC) (Shimadzu Corp., Japan) equipped with a split/splitless injector and FID detector. The samples were extracted for fatty acids from the rumen fluid before injection into the GC by dissolving them in hexane and isopropanol at a mixture ratio of 3:2 [26, 27]. The samples were extracted for fatty acids from the rumen fluid before injection into the GC by dissolving them in hexane and isopropanol at a mixture ratio of 3:2.

### Statistical analysis

This study used a completely randomized design 5 × 4, with five treatments and four replications. Observational data were analyzed using a one-way analysis of variance. Data analysis was performed using SPSS version 25.0 (IBM Corp., NY, USA). Duncan’s test considered treatment to have a significant difference at p < 0.01.

## Results

### Nutrient digestibility and rumen fermentation

The effects of *S. harbinensis LH 991* and *P. kudriavzevii B-5P* at various concentrations on nutrient digestibility and rumen fermentation are presented in Table-2. Different inoculum concentrations did not show significant differences (p > 0.01) in *in vitro* digestibility between treatments. However, in quantitative analysis, T4 had the highest nutrient digestibility compared to other treatments. T4 showed the results of *in vitro* dry matter digestibility (IVDMD), *in vitro* organic matter digestibility (IVOMD), and *in vitro* crude fiber digestibility (IVCFD) with 65.47%, 67.01%, and 68.78%, respectively, when compared with the lowest digestibility from the control with 63.76% IVDMD, 64.58% IVOMD, and 60.53% IVCFD. Meanwhile, the rumen fermentation characteristics parameters consisting of total VFA, NH_3_, and microbial crude protein (MCP) synthesis in the treatment group showed a significant improvement (p < 0.01) compared to the control. Rumen fermentation characteristics of T4 showed better results, including total VFA (166.67 mM), NH_3_ (14.00 mM), MCP synthesis (214.63 mg/100 mL), and pH rumen (6.79).

**Table-2:** Feed digestibility and rumen fermentation of experimental diets.

Parameters	Treatments					p-value

	T0	T1	T2	T3	T4	
IVDMD (%)	63.76 ± 3.34	64.30 ± 1.17	64.59 ± 0.51	64.73 ± 2.75	65.47 ± 2.51	0.516
IVOMD (%)	64.58 ± 2.75	65.88 ± 1.30	65.24 ± 0.52	65.93 ± 2.13	67.01 ± 1.04	0.135
IVCFD (%)	60.53 ± 6.80	65.45 ± 1.02	68.05 ± 4.74	68.18 ± 1.27	68.78 ± 1.85	0.283
Total VFA (mM)	113.33 ± 5.28^a^	116.67 ± 5.28^b^	121.67 ± 5.77^bc^	131.67 ± 7.64^c^	166.67 ± 5.28^d^	0.006
NH3 (mM)	11.25 ± 0.66^a^	12.17 ± 0.29^a^	12.92 ± 0.38^ab^	13.25 ± 0.90^ab^	14.00 ± 0.43^b^	0.003
MCP (mg/100 mL)	129.83 ± 0.93^a^	137.05 ± 0.04^a^	151.00 ± 0.73^ab^	171.60 ± 0.56^b^	214.63 ± 0.30^c^	0.000
pH	7.09 ± 0.09^a^	6.73 ± 0.03^b^	6.81 ± 0.02^b^	6.80 ± 0.02^b^	6.79 ± 0.02^b^	0.004

^a,b,c^Different superscripts in rows indicate highly significant differences (p < 0.01). T0=0% concentration (control), T1=1% concentration; T2=2% concentration, T3=3% concentration, and T4=4% concentration, IVDMD=*In vitro* dry matter digestibility, IVOMD=*In vitro* organic matter digestibility, IVCFD=*In vitro* crude fiber digestibility, VFA=Volatile fatty acid, NH3=Ammonia, MCP=Microbial crude protein

### SCFA composition

**Figure-1:**
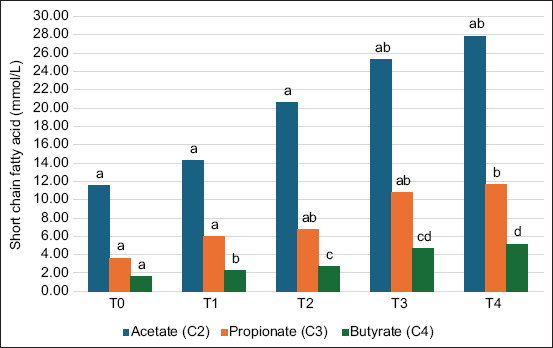
Short-chain fatty acid composition (acetate, propionate, and butyrate) of experimental diets. T0=Control (0%), T1=1% Concentration, T2: 2% Concentration, T3=3% Concentration, and T4=4% Concentration. ^a,b,c^Different superscripts indicate highly significant differences (p < 0.01).

**Figure-2:**
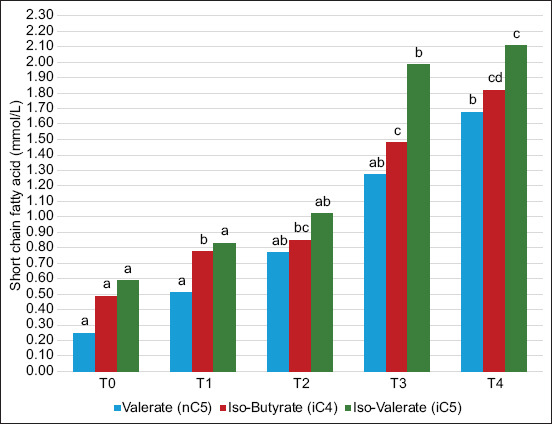
Short-chain fatty acid composition (valerate, iso-butyrate, and iso-valerate) of experimental diets. T0=Control (0%), T1=1% Concentration, T2=2% Concentration, T3=3% Concentration, and T4=4% Concentration. ^a,b,c^Different superscripts indicate highly significant differences (p < 0.01).

The results of SCFA are presented in Figures-1 and 2. The concentration of SCFAs increased in the group administrated with *S*. *harbinensis LH 991* and *P. kudriavzevii B-5P*. T4 showed that higher inoculum concentrations resulted in a higher significant (p < 0.01) SCFA composition than the control. Figure-1 shows that T4 had significantly higher (p < 0.01) proportions of acetate (27.81 mmol/L), propionate (11.61 mmol/L), and butyrate (5.16 mmol/L) compared to other treatments. Meanwhile, the SCFA results for valerate (2.11 mmol/L), iso-butyrate (1.82 mmol/L), and iso-valerate acid (1.68 mmol/L) in T4 were significantly increased (p < 0.01) compared to others (Figure-2).

## Discussion

### Nutrient digestibility and rumen fermentation

Treatment of *S. harbinensis LH 991* and *P. kudriavzevii B-5P* did not significantly affect *in vitro* nutrient digestibility, including IVOMD, IVDMD, and IVCFD (Table-2). These results need to be studied further, including the digestibility of the fiber fraction and hemicellulose content. It is important to implement the use of two strains of microbes, namely, *S. harbinensis LH 991* and *P. kudriavzevii B-5P, in vivo* in livestock and explore their mechanisms. The previous study by Jiao *et al*. [28] has reported that there were no significant differences in nutrient digestibility in this study because the microbes did not affect nutrient digestibility. Jiao *et al*. [29] reported that yeast or LAB supplementation in high-grain feed did not influence nutrient digestibility. In another study, increasing the dosage of yeast linearly increased nutrient digestibility in dairy cows [30]. The use of yeast stimulates rumen microbial protein production and the growth of anaerobic bacteria [29]. Natural microbes such as LAB and live yeast are probiotics, and supplementation can improve animal health by providing nutrients for the growth of rumen microflora and competing with pathogens [31]. Giving LAB to livestock can increase growth rates, feed efficiency, and health status by increasing cellulolytic bacteria and rumen fermentation efficiency [32]. However, the feed digestibility response to LAB or yeast supplementation remains inconsistent.

The rumen health index was determined from the pH, total VFA, NH_3_, and MCP synthesis. The rumen, as the main absorption and digestive organ, produces VFA and NH_3_ surplus in the digestive tract of ruminants [33]. The use of *S. harbinensis LH 991* and *P. kudriavzevii B-5P* resulted in significant differences in treatment using higher inoculum concentration (T4) in rumen characteristics, including total VFA, NH_3_, MCP synthesis, and rumen pH (Table-2). The rumen pH was higher in the control group than in the various treatments. The rumen pH in this study was 6–7, which indicates that it is still within the normal range for rumen health [33, 34]. A previous study by Ardani *et al*. [11] showed that the use of various strains of LAB and yeast rumen fermentation *in vitro* did not affect the rumen pH.

Meanwhile, the application of the live yeast *Saccharomyces cerevisiae* in cow rumen resulted in higher rumen pH [35, 36]. VFA production and absorption can be determined by rumen development, especially propionate and butyrate. Ma *et al*. [33] reported that yeast supplementation could regulate VFA production. Meanwhile, a report from Izuddin *et al*. [16] showed that the application of *L. plantarum* RG14 bacteria increased the VFA content in post-weaned lambs. The results of previous research are similar to this study, that is, S. *harbinensis LH 991* and *P. kudriavzevii B-5P* in the T4 increase total and individual VFA. The total VFA concentration in this study was optimal for rumen microbial growth, which was 80–160 mM [37]. There was an improvement in total VFA content in the T4 group, and it is in line with nutrient digestibility. Nevertheless, the composition of VFAs during rumen fermentation is strongly influenced by various factors, including the rumen ecology, the fermented substrate, and the microbial population [38].

NH_3_ production in the rumen originates from the degradation of food sources of protein and non-protein nitrogen (NPN) from rumen microorganism bodies. The amount of protein in the feed and its ability to decompose in the rumen affects the NH_3_ concentration [39, 40]. An increase in NH_3_ concentration agrees with the crude protein digestibility [41]. Pazla *et al*. [42] showed that NH_3_ production and absorption are directly proportional to rumen pH. NH_3_ concentration in this study is in line with previous findings where there was an increase in NH_3_ due to treatments and the highest in T4. NH_3_ in this study is optimally used for microbial protein synthesis, which requires around 4–21 mM [37]. MCP is important for supplying protein in the ruminant body, reaching 50%–80% [42]. In the present study, MCP synthesis increased in T4 cells, indicating an increase in optimal microbial protein yields to support growth. This mechanism increases MCP supply movement to the small intestine tract [36]. The results of this study align with those of Zhang *et al*. [43], who showed that activated dry yeast supplementation enhanced MCP synthesis. The balance between nitrogen and carbohydrate degradation provides an optimal environment for rumen growth [40–42].

### SCFA composition

The use of two microbial strains, *S. harbinensis LH 991* and *P. kudriavzevii B-5P*, resulted in significant differences in SCFA concentrations among treatments (Figures-1 and 2). SCFA results from the fermentation of rumen microorganisms from structural and non-structural carbohydrates. It is also constructed from the fermentation of feed and microbial proteins [37]. During fermentation, microorganisms produce various metabolites, including SCFAs, organic acids, peptides, exopolysaccharides, and enzymes. These metabolites exhibit immunomodulatory, anti-inflammatory, and antioxidant activities, proven in this postbiotic [44]. Thus far, postbiotics have been described as bioactive compounds produced from the metabolic activity of microorganisms or the fermentation process of probiotics [45]. Postbiotics are usually characterized by a longer store life and enhanced constancy because they do not contain live microorganisms. They positively affect host livestock, such as immune system control, intestinal barrier improvement, and general digestive health [46]. Wegh *et al*. [47] reported that most postbiotics come from LAB and yeast, which are produced through fermentation. Various types of strains, such as *Lactobacillus, Saccharomyces, Bifidobacterium, Faecalibacterium, and Streptococcus*, are the most common postbiotic-producing fungi and bacteria that are currently most widespread [48]. Further, yeast helps increase the growth and metabolism of bacteria by using lactate and stimulates the transformation of lactate to propionate, thereby enabling supplemented livestock to obtain more energy [49]. Malekkhahi *et al*. [50] reported that the acetate concentration was increased after the use of yeast-based feed. This was attributed to the positive influence of yeast on the growth of *D. vulgaris* and *D. desulfuricans* [51]. The increase in acetate levels in both studies was attributed to the conversion of lactate to acetic acid. [52].

Non-digested carbohydrates are fermented by rumen microflora, producing large amounts of SCFA easily absorbed by body tissues. Chen *et al*. [35] reported that SCFA is an important energy source in livestock, where butyric acid is most easily oxidized to produce CO_2._ Izuddin *et al*. [6] reported postbiotics *in vitro* experiments to investigate the impact of several levels of *L. plantarum RG14* in goat rumen on rumen characteristics, microbial populations, and gas production kinetics. Meimandipour *et al*. [53] reported that probiotics promote the growth of butyric acid-producing bacteria through a mechanism of cross-feeding. Various butyric acid-producing bacteria in the rumen also utilize acetic and lactic acid in the digestive tract [54]. Bacterial and yeast usage may impact LAB occupants in the rumen environment and influence the expansion of butyric acid-producing bacteria. Bacterial and yeast usage resulted in the highest SCFA in T4 (Figures-1 and 2). Priyankarage *et al*. [55] reported that the effect of probiotic use on SCFA concentrations was inconsistent; the administration of single-species or multi-strain multispecies microbes had no significance. However, Izuddin *et al*. [56] have shown that probiotic administration improves SCFA concentrations. This shows that administering ideal probiotics is difficult; various considerations include feed sources, probiotic strains, and interactions between probiotics and other feed additives. Energy availability is a limiting factor for microbial metabolism when bacteria utilize easily fermentable starch and carbohydrates, leading to enhanced proteolytic [56].

In this study, the use of two type of microbes, namely LAB and yeast, has shown effects on SCFA and rumen fermentation. However, the effect of probiotic use on SCFA is still inconsistent. Future studies need to explore other secondary metabolites from the use of this type of probiotic in various concentrations as a potential postbiotic.

## Conclusion

It is concluded that treatment with a concentration of 4% (T4) contained a mixture of *S. harbinensis LH 991* and *P. kudriavzevii B-5P* as LAB and yeast isolated from Budu (fermented fish) in a ratio of 50%:50% increased SCFA (acetate, propionate, butyrate, valerate, iso-butyrate, and iso-valerate acid) and rumen fermentation. Meanwhile, treatment did not affect *in vitro* digestibility.

## Authors’ Contributions

YM, LRA, and LA: Experimental design. TJS and SS: Supervised and revised the manuscript. LRA: Laboratory observations, collected data, and drafted the manuscript. YM and LA: Conducted data analysis. All authors have read and approved the final manuscript.
